# The effect a of community-based social marketing campaign on recruitment and retention of low-income groups into physical activity programmes - a controlled before-and-after study

**DOI:** 10.1186/1471-2458-12-836

**Published:** 2012-10-02

**Authors:** Janet Withall, Russell Jago, Kenneth R Fox

**Affiliations:** 1 Centre for Exercise, Nutrition and Health Sciences, School for Policy Studies, University of Bristol, Bristol, UK

**Keywords:** Social marketing, Physical activity, Exercise, Recruitment, Retention, Adherence, Community, Low income, Economically deprived

## Abstract

**Background:**

The beneficial effect of physical activity for the prevention of a range of chronic diseases is widely acknowledged. These conditions are most prevalent in low-income groups where physical activity levels are consistently lower. Social marketing is the government’s recommended approach to promoting physical activity but evidence of its effectiveness is limited. The purpose of this study was to examine the effect of a social marketing campaign on the monthly recruitment, attendance and retention levels at a community-based physical activity programme in a low income area.

**Methods:**

A six-month social marketing campaign was designed and delivered in a highly-deprived suburban neighbourhood. Analysis of variance was used to assess effects on recruitment and attendance. *χ*2 tests of independence were used to compare dropouts and adherers and effectiveness of recruitment mechanisms. Percentages were used to compare adherence rates at intervention, pre-existing sessions in the intervention area and control area sessions.

**Results:**

Attendance data were collected weekly and presented and analysed monthly to provide a view of changing participation over the six month intervention period, as compared to attendance at pre-existing sessions in the intervention area and in a control area. Recruitment into intervention sessions was significantly greater than into pre-existing and control area sessions in Month 1 (18.13v1.04 p = .007, 18.13v.30 p=.005), Month 5 (3.45v.84 p=.007, 3.45v.30 p<.001) and Month 6 (5.60v.65 p<.001, 5.60v.25 p<.001). Attendance at intervention sessions was significantly greater in all six months than at pre-existing and control area sessions; Month 1 (38.83v7.17 p<.001, 38.83v4.67, p<.001), Month 2 (21.45v6.20 p<.001, 21.45v4.00, p<.001), Month 3 (9.57v6.15 p<.001, 9.57v3.77, p<.001), Month 4 (17.35v7.31 p<.001, 17.35v4.75, p<.001), Month 5 (20.33v8.81 p=.007, 20.33v4.54 p<.001) and Month 6 (28.72v8.28 p<.001, 28.72v.4.00 p<.001). Drop-out rates in the intervention area were similar to the control area (66.2%v69.9%), and considerably lower than in pre-existing sessions (83%). In months one and two, traditional marketing techniques (posters/outdoor banners/flyers) had the greatest influence on recruitment compared to word of mouth communication (84.5%v15.5%). In months five and six word of mouth influenced 57.5% of new recruits.

**Conclusions:**

Direct comparisons with other programmes were difficult due to a lack of standard definitions of recruitment and adherence and limited reporting of findings. However when compared to pre-existing sessions and sessions delivered in a control area, monthly attendance patterns indicated that a reasonably well funded social marketing campaign increased recruitment into exercise sessions, maintained good levels of attendance and reasonable levels of adherence. Good attendance levels support on-going campaign success by offering evidence of peer and social support for the activity and increasing opportunities for social interaction. They also increase the capacity and reach of the word of mouth communication channels, the most effective form of promotion. Further study into methods of improving exercise adherence is required.

## Background

The beneficial effect of physical activity for the prevention of a range of chronic diseases is widely acknowledged [1] and public health policy in England and Wales reflects the need to increase levels of physical activity amongst the population [2]. These conditions are most prevalent in low-income groups [3] where physical activity levels are consistently lower [4].

In an attempt to tackle health inequalities and engage disadvantaged groups, the UK government has invested in improving access to, and availability of, physical activity sessions in low-income communities [5]. Evidence as to whether this increase in focus and provision is impacting the exercise habits, and thereby health, of the targeted community, is limited.

The link between physical exercise and social class is complex. Poverty can negatively affect access to facilities for exercise and physical activity [6,7], while economically disadvantaged groups are less likely to engage with physical activity interventions [8,9]. Interventions designed to change individual health behaviours are most likely to be taken up by white, middle class, females [10] while studies rarely report effects on participants from varying socioeconomic or ethnic groups [11]. Even interventions directly targeted at disadvantaged groups are less effective in engaging ethnic minority or low-income populations [12]. For example, the Walking the way to Health initiative specifically targeted those who took little exercise and/or lived in areas of poor health, yet largely recruited relatively educated and affluent participants [13].

These reviews reveal that many attempts to improve public health are unlikely to be successful in reducing health inequalities and may even have the reverse effect [10,12].

The literature relating to participation in physical activity is substantial. Factors that are consistently associated with physical activity behaviour include past exercise behaviours; perceived self-efficacy; social support; self-confidence; access to facilities; physical environment, gender and socio-economic status [14-16]. Motivations to engage in physical activity often relate to physical and mental health, weight management and fitness, enjoyment and socialising [15,17].

Common barriers to physical activity are concerns about physical health; being overweight, too old or not healthy enough to participate; low confidence and low self-efficacy; lack of enjoyment, time or motivation; a lack of suitable facilities; no-one to be active with; or insufficient disposable income [14,17-20]. Enabling factors that have been shown to support engagement in physical activity include self-confidence and self-efficacy. Social support, particularly amongst women, has also been shown to be an important influence [9,14,20].

The effectiveness of recruitment and retention strategies affect the success of every field intervention designed to reduce health inequalities, yet limited research is available to guide the practitioner. Much of the existing literature has focused on the mechanisms of recruitment into research trials, often restricted to the difficulties of increasing representation of ethnic minority groups [21]. Few studies have examined how participants, particularly those from low-income groups, might be effectively recruited into health promotion programmes. Where such public health focused research does exist, researchers rarely report their recruitment strategies or levels of success [22].

UK government policy currently recommends the use of social marketing techniques in the field of health promotion and this approach has shown some success in recruiting participants into physical activity [23,24]. However the social marketing research base is negatively impacted by some inaccurate classification of social marketing, poor study design, a lack of sophisticated analysis and evaluation and limited publication of results [25].

‘Social marketing is the adaptation of commercial marketing technologies to programs designed to influence the voluntary behaviour of target audiences to improve their personal welfare and that of the society of which they are a part’ [26].

According to this widely accepted definition from Andreasen, social marketing utilises a similar approach to behaviour change to that employed in commercial marketing. It requires a focus on consumer research; segmentation and targeting; the marketing mix (product, price, place and promotion); exchange; and competition [26]. This pilot study was designed to examine the effect of a social marketing campaign on the monthly recruitment, attendance and retention levels at a community-based physical activity programme in a low income area.

## Methods

The study used a controlled before-and-after study design, based on the TREND framework [27]. The intervention consisted of a 6-month, social marketing campaign costing £8,000, delivered between late September 2010 and March 2011. A group of nine local residents and local community and health workers acted as an advisory group to the study. They were consulted on a quarterly basis and at key points of the campaign. No intervention occurred in the control area other than normal delivery of existing sessions. A range of physical activity sessions was already available at the leisure centre in the intervention area. These activities were unrelated to the intervention and were running prior to it beginning; they are referred to here as pre-existing sessions. Data were collected at baseline, mid-point and at the end of the intervention at the five weekly intervention sessions, at baseline in the five weekly physical activity sessions running in the control area and at the nine weekly pre-existing sessions in the intervention area.

### Setting

The intervention area was Southmead, and the control area, Filwood, both suburbs of Bristol, UK. The two areas have similar population levels (11,000-12,000) with 5.2% and 3.8% Black Minority Ethnic (BME) residents respectively. Both have low life expectancy (75.3 years and 76 years respectively) compared to the UK average of 80 years [28]. The areas have similar Index of Multiple Deprivation scores (69.60, 69.23), based on income, crime, employment, health and education statistics [29]. They are the second and third most deprived wards in Bristol [30] and despite priority investment, have below average percentages of residents who exercise at least once a week [31].

### Components of the Campaign

The intervention was designed to comply with the National Social Marketing Centre’s Benchmark Criteria [32]. Formative research was conducted, the results of which have already been published [15,16]. Briefly, this work suggested that there are some key issues relating to engagement in physical activity sessions that have a greater impact on low-income groups than the general population. Issues of particular importance are cost and childcare; communication/session awareness; the social support particularly required by women to attend organised exercise sessions; the importance of socialising, fun and enjoyment; and concerns regarding perceived competence.

A summary of the input from the formative research into campaign planning is shown in Table 1.

**Table 1:** Summary input from formative research into campaign planning

Survey of session participants [16]	Focus on attracting local participants required
	Community-developed sessions most successful in terms of participation
	Dance sessions potentially popular approach to increasing participation
	Interest, enjoyment and socialising key to retention
	A mechanism to increase awareness and complement and amplify word of mouth is required
	Current promotional activities largely limited to informational fliers and poster
Interviews with session deliverers and non-participants [15]	Levels of awareness of health benefits of exercise are high
	Cost and childcare stated as practical barriers to participation
	Low session awareness amongst the target audience
	Different motivations for activity initiation (weight loss, physical and mental health, fitness) and activity maintenance (fun, interest and sociability)
	Specific social support required by most women to attend organised exercise sessions (attending with a friend)
	Issues of perceived competence particularly in comparison to other session attendees
	Application of exchange theory required to enhance the attractiveness of exercise and increase its priority so combating issues of lack of time

### Market segmentation

Social marketing prescribes that a target market should be defined in terms that relate to behaviour. The target market selected comprised of individuals with an awareness of the benefits of exercise and positive attitudes towards it; anxiety-related barriers to joining exercise sessions; a fear of arriving alone (lack of relatedness); and a perceived lack of competence and autonomy. The formative research indicated that this target group was likely to be substantial in size and largely female.

The intervention marketing mix and branding strategy are detailed below.

### The product

The product development was based on findings from the formative research, and the literature relating to recruitment and retention. Three kinds of dance session (Line Dancing, Zumba and Salsa), an instructor-led gym session, and Body Tone, a balance and stretching session, were selected to form the product offering [15]. These five sessions each took place once per week at 9.15 am, so a different session ran every weekday morning. Sessions were all held at the local leisure centre, which is described under ‘Place’ below. It was hoped that parents would attend sessions immediately after ‘the school run’, prior to returning home and engaging with their established morning routine. Socialising is associated with adherence to exercise, so free tea and coffee was available before and after sessions and socialising was actively encouraged [15,17].

### Branding strategy

Branding is central to social marketing. It builds strong bonds between the product and its target market by creating a tone, feeling or emotional response to a product or service [33]. A number of brand names were tested with the target group. Fit and Fab was selected because fitness was a commonly used term and was considered less ‘worthy’ than health or exercise. Using ‘Fab’ with ‘Fit’ diluted its seriousness, suggested an inclusiveness of women, was not associated with slimness and was easy to remember and pass on.

**Figure 1:**
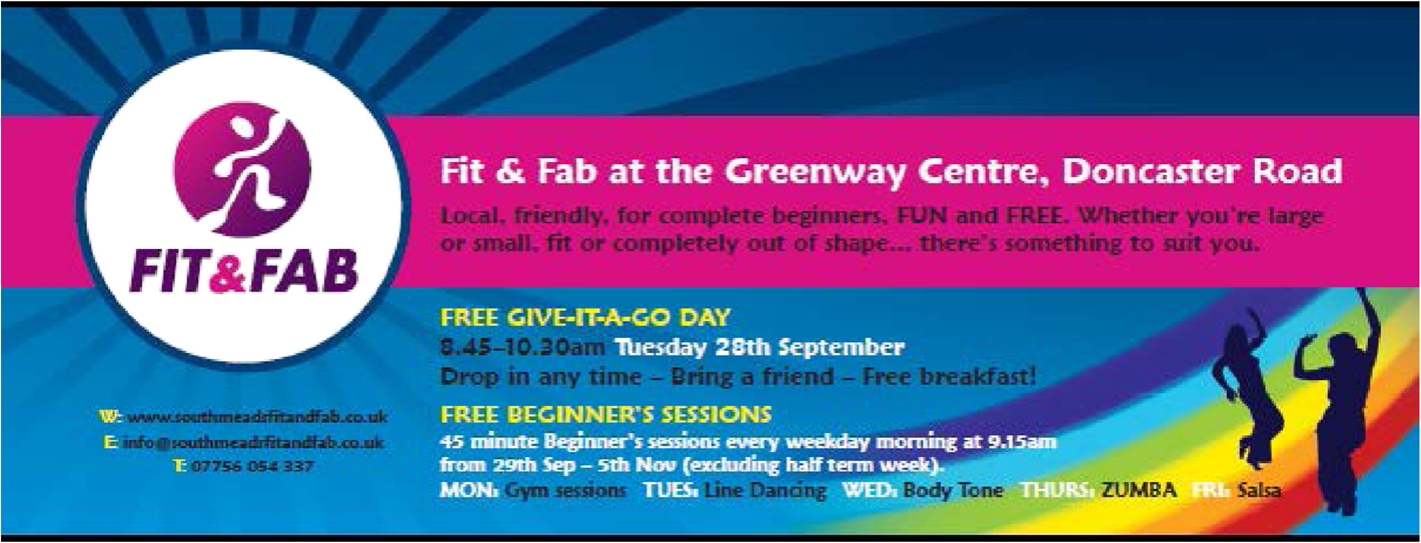
Fit and Fab Outdoor banner (8ft x 3ft).

The branding strategy was designed to generate the feeling of a local movement, to create momentum, convey a sense of fun and enjoyment and to imply that this was a programme for ‘people like me’. It emphasised session accessibility for beginners, the unfit, the overweight and people of any age. Branding approaches were tested with the advisory group and members of the target group. The final Fit and Fab logo is shown in Figure 1.

All the five sessions were branded as Fit and Fab. The intention was to create one offering within which participants were offered choices. The different sessions were the product mechanisms which delivered the brand values of localism, accessibility, fun, enjoyment and social norm and which enabled the intervention’s overall objective (increased participation in physical activity) to be achieved.

### People, Place and Price

Session leaders were encouraged to maximise fun and enjoyment. They were issued with a session delivery guide and provided with regular feedback.

Fit and Fab was delivered in an old school building which was converted into a leisure centre 25 years ago. It is run by a charitable trust for the benefit of local residents and is geographically close to a large proportion of the intervention area’s population.

The price of a product is ‘the cost that the target market associates with adopting the new behaviour’ [33]. This incorporates both monetary and non-monetary costs, such as time, energy and any psychological costs [34]. An effective pricing strategy tips the balance between the costs and benefits of the product such that the target market chooses to exchange their current behaviour for the target behaviour. Monetary cost is frequently presented as a barrier to engaging in organised physical activity and has a proportionately greater impact on low-income groups [35]. While some argue that free activities are less valued [34], the impact of a very low cost approach in a low-income area has not been fully tested. For the first six weeks of this intervention all sessions were free, from then on the cost was £1.

Issues of time, and fitting exercise into the daily routine, were often cited as barriers to exercising in the formative research. As a result a 9.15 am start time and short 45 minute sessions were used. As tackling issues of childcare was impractical for this campaign, those with children of at least school age were targeted.

Issues of low self-esteem, confidence to exercise and confidence to attend an activity session were addressed though the promotional campaign.

### Promotion

Promotion is the development and deployment of persuasive materials and activities to convincingly communicate the product benefits and its value, particularly in relation to competing products or activities [33,34]. The key campaign promotional messages emerged from the analysis of barriers, enablers and motivations to change behaviour.

The selection of media channels was based on the target group’s media habits, the communication objectives, the available local media and their geographic coverage, and budget constraints. Promotional activities were designed not to spill over into neighbouring, affluent areas to avoid the usual bias towards engaging the middle classes [13]. As Fit and Fab was previously unknown, and the intention was to move the consumer rapidly towards action, the promotional campaign aimed to reach the target group a minimum of three times, preferably more. The key promotional elements included 17 outdoor banners (8 ft × 3 ft); 3 door drops (4,500 residences each drop); street leafleting; leaflet distribution via schools, community groups and GPs; a poster campaign; face to face recruitment; local press; a campaign blog; a text campaign; two taster sessions; and a loyalty scheme.

The main promotional objectives were to build high levels of awareness, tackle barriers and support engagement, promote the Fit and Fab brand and its values, generate word of mouth promotion, create a ‘buzz’ around the area and offer session trialability.

**Figure 2:**
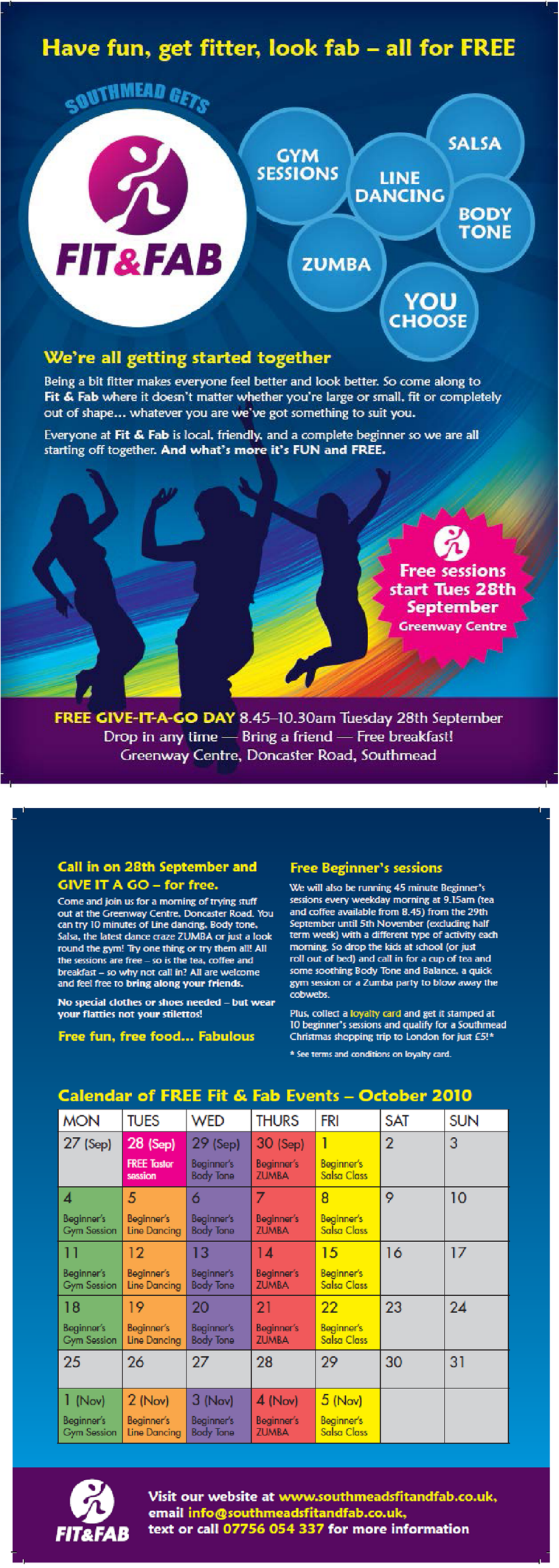
Flyer – launch version.

Fit and Fab materials are shown in Figures 1 and 2 and the campaign budget is detailed in Table 2.

**Table 2:** Social marketing campaign budget

**Item**	**Cost**
Design and branding	1600
Flyers	700
Banners	750
Posters	390
On site display and signage	295
Loyalty scheme	505
Leaflet distribution and delivery	880
Text campaign	80
Campaign launch event	485
Subsidy for free and £1 sessions (leaders and room hire)	1710
Blog design, development and hosting	90
**Total**	**7395**

During the period of the intervention the pre-existing and control area sessions were promoted through posters at the session venues and inclusion in the programme of events published by each venue. Details of the pre-existing sessions in the intervention venue were included in a flyer that was delivered to c4,500 homes in the intervention area.

### Procedures

The data collection, analysis and results were conducted in four main sections, participants, recruitment, attendance and adherence.

Participant data were collected to assess equivalence across the intervention and control areas. Attendance data enabled assessment of the effect of the campaign on participant recruitment, attendance and adherence in the intervention and pre-existing sessions, and as compared to the control area. Attendance data were collected weekly and presented and analysed monthly. This study was designed to mirror usual community practice; as such participants were allowed to join the sessions throughout the intervention. Therefore monthly data analysis was considered to be the most informative method of presenting the results. This method provided an overview of participation patterns across the intervention period rather than just at a beginning and end point, and enabled the calculation of adherence rates. Data from the participant surveys were collected to enable a comparison of the characteristics of pre-existing attendees and those recruited through the campaign, and to compare the characteristics and motivations of adherers and drop outs.

### Data collection

The intervention site was a local leisure centre. It offered a range of physical activity sessions and operated a gym. The leaders of these pre-existing sessions, and the gym manager at the leisure centre, agreed to provide their attendance data. All session leaders and the gym manager at the leisure centre and health park in the control area also agreed to provide their session attendance data.

To provide baseline data, attendance data at these pre-existing sessions in the study area (nine sessions per week), and sessions in the control area (five sessions per week across two centres) were collected for one month prior to the intervention and for the whole of the intervention period. Attendance data at all the intervention (Fit and Fab) sessions were collected using a paper-based register, for the whole of the intervention period.

Three different questionnaires were used to collect demographic data and reasons for participation from five different participant groups.

Group 1 were participants at physical activity sessions already running at the leisure centre where the intervention was conducted (pre-existing sessions). At baseline, Group 1 completed Questionnaire A which recorded name, age, gender, postcode, height, weight, ethnicity, attendance duration, attendance frequency, attendance with a friend, confidence to start attending alone, reasons for attending and awareness of the intervention.

Group 2 were participants in physical activity sessions in the control area. They also completed Questionnaire A at baseline, as described above. Group 3 were participants in the intervention (Fit and Fab) sessions during month 1 and they completed Questionnaire A on recruitment.

Group 4 were those who had participated in the intervention (Fit and Fab) from month 1 and were still attending after 3–4 months. During months 3 and 4 they completed Questionnaire B. This recorded name, age, gender, postcode, height, weight, ethnicity, attendance duration, attendance frequency, attendance with a friend and investigated reasons for adherence and how this was affected by the campaign.

Group 5 were participants who were recruited into the intervention during month 6. Group 5 completed Questionnaire C on recruitment which recorded how the campaign influenced attendance, and reasons for attendance.

For recruitment into groups 1 and 2, information sheets were distributed at sessions and 47 consent forms and questionnaires were completed in the intervention area and 52 in the control area. Group 3, 4 and 5 participants were recruited at Fit and Fab sessions during month 1, months 3 and 4 and month 6 of the intervention respectively. Consent forms and the questionnaire were completed by 46 participants in month 1, 24 participants in months 3 and 4, and 41 participants in month 6.

The study was approved by the University of Bristol School of Applied Community Health Studies Research Ethics Committee (Ref 015/10).

### Analyses

Participants in pre-existing sessions in the intervention and control areas (Groups 1 & 2) were compared. Participation data from intervention, pre-existing, and control area sessions were compared to assess the effect of the campaign on participant recruitment, attendance and adherence. Recruitment, attendance and adherence rates at the different types of Fit and Fab sessions were also compared. All analyses were conducted in SPSS (version 16.0) and alpha was set at p < 0.05.

### Participant equivalence

Chi-squared tests of independence were used to examine if participants attending pre-existing physical activity programmes in the intervention and control areas differed by age category, BMI range, ethnicity, attendance frequency, attendance with a friend or confidence to attend alone.

### Definitions

There are no widely accepted definitions of the terms recruitment, retention or adherence (retention and adherence appear to be largely interchangeable), particularly relating to public health interventions. Editors appear to be reluctant to publish articles where recruitment and adherence are the prime focus [36], and details of recruitment and adherence procedures are often not reported in published studies [22]. In this study recruitment reflects whether the target group overcame any pertinent barriers and were sufficiently motivated to engage with the intervention at least once. Attendance is the number of participants at any one session, and indicates the scale of the impact of the intervention.

In most published studies, adherence has been defined as attendance at a certain percentage of the available sessions. Due to the extended length of this intervention, the large number of sessions available and the on-going recruitment throughout the intervention, this approach could have led to misleading results. In this study, two levels of adherence were set. Both required current, on-going participation, defined as at least one attendance during the final month of the intervention. In addition, ‘Low adherence’ required 6–12 session attendances over the 6 months of the intervention, and ‘High adherence’ >12 attendances. The total number of adherers is defined as the sum of the low and high adherers.

### Recruitment and attendance

To compare recruitment and attendance rates between the Fit and Fab, pre-existing and control area sessions, analysis of variance tests were used with mean monthly recruitment rates or mean monthly attendance rates as the outcome and session type (intervention/pre-existing/control) as a factor. Significant main effects were further explored using Bonferroni post-hoc multiple comparisons. In addition, *χ*2 tests of independence were used to compare the relative importance of different recruitment mechanisms in the early and late stages of the intervention.

### Adherence

Percentages were used to compare adherence rates at Fit and Fab, pre-existing and control area sessions, and between the different types of Fit and Fab session. Additionally, *χ*2 tests of independence were used to examine if intervention drop-outs and adherers, differed by age category, gender, postcode, BMI range, ethnicity, attendance duration, attendance frequency, attendance with a friend or motivations to participate.

## Results

The results are presented in four main sections, participants, recruitment, attendance and adherence. Attendance data are reported monthly to provide a view of changing participation over the six month intervention period. Total numbers of participants, attendees and adherers in the five Fit and Fab sessions, the five control area sessions and the nine pre-existing sessions in the intervention area were compared. The sessions were grouped together as the attendance numbers in the two latter groups were too small to facilitate individual analyses.

### Participant equivalence

There were differences in the age ranges of participants attending pre-existing physical activity programmes in the intervention and control areas (*χ*2 = 16.02, df = 6, p = 0.014), with sessions in the intervention area attracting more under 25 year olds (27.7% v 20.8%) and over 54 year olds (40.4% v 26.4%) and fewer 25–54 year olds (31.9 v 52.9%). There were also differences in ethnicity with fewer BME participants in the control area (*χ*2 = 7.97, df = 3, p = 0.047).

### Recruitment

Analysis of variance showed differences in recruitment rates in October (F = 10.547, p = .003), February (F = 35.972, p < .001) and March (F = 39.100, p = .001). Bonferroni post-hoc multiple comparisons showed that Fit and Fab sessions had higher monthly recruitment rates than either pre-existing or control area sessions in October (18.13 v 1.04 p = .007, 18.13 v .30 p = .005), February (3.45 v .84 p = .007, 3.45 v .30 p < .001) and March (5.60 v .65 p < .001, 5.60 v .25 p < .001). Details are shown in Table 3. Chi-squared tests of independence showed variation in the effects of different communications channels on recruitment into the intervention (*χ*2 = 21.9, df = 4, p < 0.001).

**Table 3:** Comparison of mean weekly recruitment (expressed for each month) at intervention, pre-existing and control sessions

	**Recruitment to fit and fab sessions mean (SD)**	**Recruitment to pre-existing sessions mean (SD)**	**Recruitment to control area sessions mean (SD)**	***F- values***	***Df***	***p values***
Sept 2010	na	.86 (1.10)	1.28 (1.46)	*F = 1.636*	*1*	*.206*
Oct 2010	18.13 (12.30) ^a, b^	1.04 (.42) ^a^	.30 (.41) ^b^	*F* = 10.547	*2*	*.003*
Nov 2010	2.25 (1.68)	.78 (.41)	.31 (.38)	*F* = 3.908	*2*	*.060*
Dec 2010	1.00 (.35)	.78 (.61)	.08 (.14)	*F* = 3.493	*2*	*.089*
Jan 2011	3.53 (3.03)	1.29 (.43)	.38 (.595)	*F* = 3.527	*2*	*.080*
Feb 2011	3.45 (.81) ^a, b^	.84 (.26) ^a^	.25 (.50) ^b^	*F* = 35.972	*2*	*<.001*
Mar 2011	5.60 (1.69)^a, b^	.65 (.25) ^a^	.25 (.29) ^b^	*F* = 39.100	*2*	*.001*

Superscripts (^a, b, c^) indicate mean difference in Bonferroni comparisons (p < 0.05).

^a^ = Fit and Fab v pre-existing sessions, ^b^ = Fit and Fab v control area sessions, ^c^ = pre-existing sessions v control area sessions.

In months one and two, traditional marketing techniques (posters/outdoor banners/flyers) had the greatest influence on recruitment compared to word of mouth communication (84.5%v15.5%). In months five and six word of mouth influenced 57.5% of new recruits. The results of the analysis are shown in Table 4.

**Table 4:** Communications mechanisms reported as important to recruitment in early and late stages of the intervention (n = number of people reporting each factor as an influence)

	**Months 1 and 2 n (column %)**	**Months 5 and 6 n (column %)**	***χ2, df***	***p values***
Posters	25 (35.20)	7 (17.50)	21.938, 4	*<.001*
Outdoor banner	15 (21.10)	3 (7.50)		
Word of mouth	11. (15.50)	23 (57.50)		
Doordrop leaflet	15 (21.10)	6 (15.00)		
Leaflet from child’s school	5 (7.00)	1 (2.50)		

Analysis of variance tests on the mean monthly recruitment rates at the five different types of session (Gym, Line Dancing, Body Tone, Zumba, Salsa) showed differences in recruitment rates in February (F = 12.415, p < .001) and March (F = 7.808, p = .001). Bonferroni post-hoc multiple comparisons showed that Zumba had higher monthly recruitment rates than all other sessions in February (7.5 v 1.5 p < .001, 7.5 v 2.0 p = .001, 7.5 v 1.75 p < .001, 7.5 v 4.0 p = .035) and March (13.4 v 5.0 p = .030, 13.4 v 1.8 p = .001, 13.4 v 2.6 p = .002, 13.4 v 6.0 p = .049). The results are shown in Table 5.

**Table 5:** Comparison of mean weekly recruitment (expressed for each month) between different types of intervention sessions

	**Gym session mean (SD)**	**Line dancing mean (SD)**	**Body tone mean (SD)**	**Zumba mean (SD)**	**Salsa mean (SD)**	***F- values***	***df***	***p values***
Oct 2010	15.00 (14.93)	19.0 (17.35)	14.5 (15.09)	19.75 (10.15)	14.75 (9.47)	*F* = .135	*4*	*.970*
Nov 2010	1.40 (1.14)	1.20 (2.68)	2.25(2.22)	3.25 (2.99)	2.75 (1.50)	*F* = .701	*4*	*.602*
Dec 2010	.50 (.71)	1.00 (1.41)	1.00 (1.00)	1.00 (1.00)	1.67 (.577)	*F* = .490	*4*	*.744*
Jan 2011	4.00 (2.94)	1.67 (1.16)	3.33 (4.16)	5.33 (6.66)	3.00 (2.65)	*F* = .364	*4*	*.829*
Feb 2011	1.50 (1.00) ^c^	2.00 (1.83) ^f^	1.75 (.96) ^h^	7.50 (1.73) ^c, f, h, j^	4.00 (1.41) ^j^	*F* = 12.415	*4*	*<.001*
Mar 2011	5.00 (2.16) ^c^	1.80 (2.49) ^f^	2.60 (2.30) ^h^	13.40 (5.94) ^c, f, h, j^	6.00 (3.74) ^j^	*F* = 7.808	*4*	*.001*

Superscripts (^a-j^) indicate mean difference in Bonferroni comparisons (p < 0.05).

^a^ = Gym v Line Dancing, ^b^ = Gym v Body Tone, ^c^ = Gym v Zumba, ^d^ = Gym v Salsa, ^e^ = Line Dancing v Body Tone, ^f^ = Line Dancing v Zumba,

^g^ = Line Dancing v Salsa, ^h^ = Body Tone v Zumba, ^i^ = Body Tone v Salsa, ^j^ = Zumba v Salsa.

### Attendance

The total number of individuals who attended at least one session over the six month intervention was 364. In total these 364 participants attended 2,618 Fit and Fab sessions. In the intervention area there were 3,553 females, aged 20–74 years, so this equates to a conversion rate of 10.24%.

**Figure 3:**
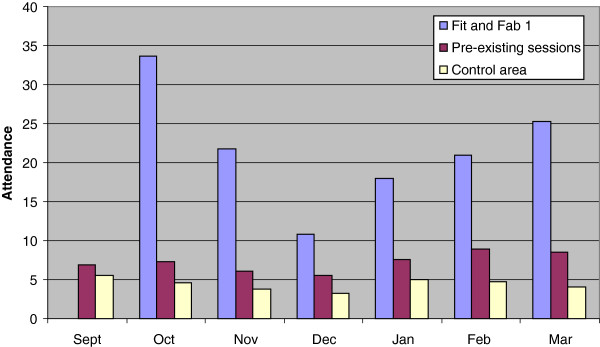
Mean weekly attendances at Fit and Fab, pre-existing and control area sessions.

Bonferroni post-hoc multiple comparisons showed that Fit and Fab sessions had higher monthly attendance rates than either the pre-existing or control area sessions, in all 6 months of the intervention; October (38.83 v 7.17 p < .001, 38.83 v 4.67, p < .001), November (21.45 v 6.20 p < .001, 21.45 v 4.00, p < .001), December (9.57 v 6.15 p < .001, 9.57 v 3.77, p < .001), January (17.35 v 7.31 p < .001, 17.35 v 4.75, p < .001), February (20.33 v 8.81 p = .007, 20.33 v 4.54 p < .001) and March (28.72 v 8.28 p < .001, 28.72 v .4.00 p < .001). There was also a difference between attendance rates in the pre-existing and control area sessions in December (6.15 v 3.77 p = .003). These results are shown in Figure 3.

Bonferroni post-hoc multiple comparisons showed that Zumba sessions had consistently higher monthly attendance rates than other sessions, excluding Salsa. Salsa had higher monthly attendance rates than some other sessions in February and March. The results of the analysis are shown in Table 6.

**Table 6:** Comparison of mean weekly attendance (expressed for each month) between different types of intervention sessions

	**Gym session mean (SD)**	**Line dancing mean (SD)**	**Body tone mean (SD)**	**Zumba mean (SD)**	**Salsa mean (SD)**	***F- values***	***df***	***p values***
Oct 2010	26.67 (5.51) ^c^	37.33 (1.53)	28.25 (7.93) ^h^	42.50 (6.76) ^c, h^	33.50 (3.87)	*F* = 4.594	*4*	*.016*
Nov 2010	16.20 (3.90) ^c^	19.40 (7.57)	18.00 (3.92)	31.50 (11.12) ^c^	24.00 (2.45)	*F* = 3.731	*4*	*.023*
Dec 2010	8.00 (1.41)	9.5 (3.54)	6.00 (1.00) ^h^	14.00 (0.00) ^h^	10.67 (2.31)	*F* = 7.740	*4*	*.007*
Jan 2011	17.25 (3.86)	14.33 (2.08) ^f^	13.67 (3.51) ^h^	23.33 (2.31) ^f, h^	21.67 (2.52)	*F* = 6.090	*4*	*.008*
Feb 2011	14.75 (3.54) ^c,d^	18.50 (1.73) ^f^	12.50 (3.32) ^h,i^	34.00 (2.00) ^c, f, h^	25.25 (2.63) ^d,i^	*F* = 20.263	*4*	*<.001*
Mar 2011	19.00 (2.71) ^c,d^	21.00 (2.55) ^f,g^	17.20 (2.39) ^h,i^	48.80 (7.26) ^c, f, h^	40.00 (5.15) ^d,g, i^	*F* = 48.677	*4*	*<.001*

Superscripts (^a-j^) indicate mean difference in Bonferroni comparisons (p < 0.05).

^a^ = Gym v Line Dancing, ^b^ = Gym v Body Tone, ^c^ = Gym v Zumba, ^d^ = Gym v Salsa, ^e^ = Line Dancing v Body Tone, ^f^ = Line Dancing v Zumba,

^g^ = Line Dancing v Salsa, ^h^ = Body Tone v Zumba, ^i^ = Body Tone v Salsa, ^j^ = Zumba v Salsa.

### Adherence

Total adherence rates at Fit and Fab sessions are similar to those in the control area but with proportionately fewer high adherers (total adherers 33.8% v 30.1%, high adherers 13.2% v 17.0%). Total adherence rates at the pre-existing sessions in the intervention area were lower than those at the intervention sessions, but with a more even distribution between low and high adherers (total adherers 33.8% v 18.6%, high adherers 13.2% v 8.9%, low adherers 20.6% v 9.7%). Total adherence rates at the different Fit and Fab sessions varied. Line dancing exhibited the highest rate (38%), followed by Salsa (25%), while Zumba had the lowest levels of adherence (17%).

There were differences in adherence by age range (*χ*2 = 11.3, df = 5, p = .046), with more 25–34 and 45–54 year olds dropping out than adhering (34.6% v 18.9%, 36.9% v 5.4%) and more 35–44, 55–64 and 65+ year olds adhering than dropping out (37.8% v 5.4%, 18.9% v 11.5%, 13.5% v 3.8%). There were also differences in attendance duration between adherers and non-adherers (*χ*2 = 10.90, df = 2, p = .006) with non-adherers more likely to have attended for less than 1 month (85.2% v 47.4%) and adherers more likely to have attended for 1–3 months (44.7% v 14.8%). Attendance duration is relatively low in both groups because participants were surveyed during the first 3–4 months of the intervention. The characteristics of adherers and non-adherers are compared in Table 7.

**Table 7:** Characteristics of adherers at intervention (Fit and Fab) sessions (n = 38) and non-adherers (n = 27)

**Characteristics**	**Adherers at Fit and Fab sessions n (%)**	**Non-adherers at Fit and Fab sessions n (%)**	***χ2, df***	***P value***
**Gender (n = 65)**				
Male	1 (2.6)	2 (7.4)	.82, 1	.366
Female	37 (97.4)	25 (92.6)		
**Age (n = 63)**				
<18 years	0 (0.0)	0 (0)	11.3, 5	.046
18–24 years	2 (5.4)	2 (7.7)		
25–34 years	7 (18.9)	9 (34.6)		
35–44 years	14 (37.8)	4 (15.4)		
45–54 years	2 (5.4)	7 (26.9)		
55–64 years	7 (18.9)	3 (11.5)		
65 years +	5 (13.5)	1 (3.8)		
**BMI (n = 59)**				
Underweight (<18.5)	2 (5.9)	0 (0.0)	5.1, 3	.166
Normal weight (18.5-24.9)	15 (44.1)	13 (52.0)		
Overweight (25–29.9)	15 (44.1)	7 (28.0)		
Obese (30+)	2 (5.9)	5 (20.0)		
**Ethnicity (n = 65)**				
White	28 (73.7)	17 (63.0)	1.9, 3	.597
Black/Afro-Caribbean	2 (5.3)	4 (14.8)		
Asian	7 (18.4)	5 (18.5)		
Other	1 (2.6)	1 (3.7)		
**Postcode (n = 65)**				
BS10	27 (71.1)	18 (66.7)	.14, 1	.706
Non-BS10	11 (28.9)	9 (33.3)		
**Attendance duration (n = 65)**				
Less than 1 month	18 (47.4)	23 (85.2)	10.9, 2	.006
1–3 months	17 (44.7)	4 (14.8)		
4–6 months	3 (7.9)	0 (0.0)		
**Attendance frequency (n = 65)**				
First time	6 (15.8)	10 (37.0)	5.6, 1	.060
Every week	32 (84.2)	16 (59.3)		
Every other week	0 (0.0)	0 (0.0)		
Once a month	0 (0.0)	0 (0.0)		
Now and again	0 (0.0)	1 (3.7)		
**Attend with a friend (n = 63)**				
Yes	20 (52.6)	14 (56.0)	.07, 1	.793
No	18 (47.4)	11 (44.0)		

## Discussion

Overall this study found that monthly attendance patterns at a campaign based on a social marketing framework indicated that a reasonably well funded social marketing campaign increased recruitment into exercise sessions, maintained good levels of attendance and reasonable levels of adherence.

Direct comparisons with other approaches were difficult due to a lack of standard definitions of recruitment and adherence and little detailed reporting of findings.

A comparison of physical activity participants in the control and intervention areas prior to the intervention was designed to establish whether the control area was sufficiently similar to the intervention area to offer a valid control for the intervention. The findings showed that while participants from both areas were mainly white the intervention area had engaged more Black Minority Ethnic (BME) participants in existing exercise sessions. However, the target for the intervention recruitment strategies was a segment of the general population, not existing exercisers, and the BME populations in these groups were comparable (5.2% v 3.8%).

The ages of those who were already participating in exercise sessions also differed, with the intervention area attracting less middle-aged participants. This difference was not reflected in the general population of the two areas where the average ages were 35.89years (intervention area) and 34.38years (control area), with a similar percentage (37.0% v 38.1%) in the middle age ranges (30–59yrs).

It is difficult to identify a directly comparable control group in any community based and/or social marketing intervention, yet studies that offer better levels of evidence than before and after designs are needed [37-39]. As such best efforts were made to select an intervention area on the basis that a control area offering good comparability was available. At population level the control and intervention areas in this study were very similar [40].

### Recruitment

Fit and Fab sessions had considerably higher monthly recruitment rates than either the pre-existing or control area sessions in October, February and March. The main thrust of the Fit and Fab recruitment campaign occurred in late September and early October, with a second phase in mid-January. These results indicate that the campaign did affect recruitment in October and February. The on-going increase in recruitment into March may have been an extension of the effect of the January phase of the campaign. However, the substantial increase in the influence of word of mouth on recruitment between the early and late phases of the intervention (see Table 4), indicates recruitment could have been boosted due to increased promotion via this channel.

Research supports the finding that WOM has a greater influence on behaviour than any marketer controlled promotional tool [41,42]. Here the relatively large numbers of participants increased the social networks available as communications channels, and so increased the likelihood of non-participants hearing about Fit and Fab from multiple sources. This helped in creating a sense of a socially acceptable norm [43].

Zumba, a Latin inspired form of dance-based exercise, was relatively new to the UK; however it exhibited higher monthly recruitment rates than any other session during February and March. It is possible that the ‘newness’ of the activity may have generated interest amongst those who had previously dismissed participation in commonly available activities. This session generated the most unprompted positive comments on the survey documents. This high level of enthusiasm is consistent with the theory of word of mouth communication, in that consumers with either very positive or very negative views are much more likely to communicate their opinions than those with moderate views [42,44,45]. Customer ‘delight’ is considered to be the central driver of positive output WOM. That the session that generated the most overt enthusiasm was most impacted by positive output WOM is well supported in the literature [41,44].

It is necessary to consider that attendance at Fit and Fab sessions may have been as a result of cannibalisation of other, more expensive sessions in the area. While it is not possible to rule this out, the results show that there was no drop in recruitment or attendance at pre-existing sessions when Fit and Fab started. In fact, compared to September both recruitment and attendance at pre-existing sessions increased in October (the first month of the intervention). In addition, none of the pre-existing sessions took place during the day; they all ran in the evening and at weekends and so largely targeted a different audience.

### Attendance

Attendance at Fit and Fab sessions was substantially higher than at both the control area and pre-existing sessions over the whole intervention. The latter two had fairly low average attendance levels. These were largely non-commercial activities supported by the local authority or a charitable trust to try and improve health in these two low income areas. These low attendance levels illustrate the challenge facing those attempting to increase participation in physical activity in such areas.

Fit and Fab showed a conversion rate of 10.24%. It is very difficult to find similar studies with which to compare this. A review of the Health Education Board for Scotland’s Active Living mass media campaign showed a 0.1 – 1% response rate [46]. One research study mailed households in a low-income area in Scotland offering a fitness assessment or an exercise consultation. A 12.3% response rate, with 10.4% actually attending a session was regarded as ‘good’ [47]. In the commercial sector published rates vary considerably. The Royal Mail quotes an average 5% response rate to a door drop [48] while the Direct Marketing Association’s 2010 Response Rate Trend Report claims letter-sized envelopes have a 1.38 percent response rate [49]. These figures relate to responses or enquiries, which are not necessarily converted to sales. In this context the Fit and Fab response rate could be described as very good.

The consistently high levels of attendance may have been important in providing tangible evidence of the clear social and community support for the activity. This may have helped build a sense of relatedness in that the community were getting together in some numbers. It may also have avoided the negative impact of small attendances where participants feel exposed and unsupported by peers.

### Adherence

A lack of a common definition and clear published adherence statistics means that studies testing different approaches to improving adherence cannot be compared. However, based on a number of studies where again definitions were not specified, James claims that 40 to 65% of individuals initiating exercise programmes are predicted to drop out within 3 to 6 months [37,38,42]. In a study of health clubs 69.8% of members were retained for at least 36 weeks and 60.6% for 12 months. However, paying membership fees does not necessarily equate to attendance and of those who were still members at 6 months 44% visited less than once a week [50]. A review of exercise referral schemes reported drop-out rates of between 58% and 88% over a 10–12 week programme [51]. Overall these findings do support the commonly acknowledged difficulties associated with maintaining physical activity [52].

Fit and Fab’s drop-out rate was similar to that in the control area (66.2% v 69.9%), and considerably lower than in the pre-existing sessions (83%). Total adherence rates at the different Fit and Fab sessions varied. Line dancing exhibited the highest level of adherence (38%), followed by Salsa (25%), while Zumba, had the lowest levels of adherence (17%). Line dancing attracted older participants and the age patterning of adherence showed 32.4% of adherers were older participants (55 years+). Zumba was largely previously untried by participants so it was unsurprising that above average numbers of participants found it did not suit them.

There is literature that indicates that high levels of attendance in the early phase of exercising establishes an exercising habit and increases the probability of adherence [41,50]. This supports the finding here that if participants attended for more than one month they became much more likely to adhere. The intervention’s initial six week period of free sessions supported the development of a habit to increase the likelihood of long term adherence.

Of all the factors affecting motivation to exercise, enjoyment was the only one which differed between adherers and non-adherers. This finding is supported by the literature that associates enjoyment with adherence to physical activity [53-55]. It also indicates that, even prior to exercising, a belief that the activity will be enjoyable is linked to adherence.

Although there is a lack of evidence in the area it is possible to hypothesise that there are a number of practical implications for policy makers from this study. Investment in promotion, sufficient that the target market is exposed multiple times to good quality materials and well-designed messages, is important if high levels of recruitment are to be achieved, and therefore good levels of WOM generated. While it is widely accepted that enjoyment is associated with exercise adherence, policy makers should consider that in order to activate output WOM, the most influential marketing tool, levels of enjoyment need to be very, not just moderately, high [42,44,45]. In addition, an intervention may require a certain longevity in order to fully capitalize on the sales impact of WOM.

The findings from this study suggest that high levels of participation may have multiple benefits and should be a priority for interventions and a consideration when setting budgets. Good attendance levels increase the capacity and reach of the WOM communication channel, offer evidence of peer and social support for the activity and increase the opportunities for socialising and therefore building relatedness.

Establishing an exercise habit can combat early drop out, so techniques to promote high levels of initial engagement, such as an introductory free period may be effective [41,50]. In addition, new or novel forms of activity may generate interest amongst those who had previously dismissed participation in commonly available activities.

### Sustainability

The Fit and Fab intervention is now managed by the Southmead Development Trust, the organisation that owns the intervention venue. The trust is not a commercial enterprise and operates the leisure centre for the benefit of the local community. As such, the demands on sustainability are lower in that only the overhead costs of the room hire and the instructors employed by the trust need to be charged to the programme. However the cost of instructors paid on a session only basis must be covered. Contributions towards maintaining Fit and Fab have been made by Bristol PCT (£500) and the Southmead and Henbury Neighbourhood Renewal Fund (£1000). This funding has largely been used for additional bursts of promotion and as a subsidy for quieter periods such as Christmas and school holidays.

Although the researchers do not now have access to attendance figures the management team at the Southmead Development Trust continues to operate the Fit and Fab sessions almost two years after the intervention began. The cost is still £1. Their intention is to continue the programme.

### Limitations

Much of the existing evidence base, relating to the application of community-based social marketing techniques, uses weak design and short-term interventions. This study employs a controlled before-and-after design, the second level of evidence of intervention efficacy [38]. However, when using a control with this design any substantial differences across groups at baseline may suggest dissimilar influences and a lack of direct comparability [37]. At population level the control and intervention areas were very similar; however those who were already exercising in the two areas differed in age range and ethnicity.

Due to resource constraints drop-outs from the sessions were not followed up to discover if they were continuing to exercise. Potential adherers i.e. those who were recruited in, and attended regularly during month 6 (and possibly month 5), but whose on-going attendance patterns were not available as part of the study data, could not be included in the adherer/retained category. Also ‘lapsed’ exercisers could only be included if they returned prior to the end of month 6.

It is also important to acknowledge that the community based research design allowed for additional participants to join the sessions during the programme. This decision was taken to mirror usual community practice. While this increases the external validity of the study (i.e. the study reflects usual practice) it does mean that the internal study validity is less controlled. While this is not perfect there is always a trade-off between internal and external validity in study designs and for this study we opted to focus on external validity.

The primary focus of this study was to investigate the effectiveness of an approach to recruitment and retention into physical activity sessions. There was no measurement of physical activity levels, intensity or the biological markers of improved health (i.e. BMI, blood pressure, cholesterol levels etc.). To have incorporated such measures would have meant the study tested recruitment into a research project rather than into physical activity sessions.

## Conclusions

Direct comparisons with other programmes were difficult due to a lack of standard definitions of recruitment and adherence and limited reporting of findings. However, this study found that when compared to pre-existing sessions and sessions delivered in a control area, monthly attendance patterns indicated that a reasonably well funded social marketing campaign increased recruitment into exercise sessions, maintained good levels of attendance and reasonable levels of adherence. Good attendance levels support on-going campaign success by offering evidence of peer and social support for the activity and increasing opportunities for social interaction. They also increase the capacity and reach of the word of mouth communication channels, the most effective form of promotion. Further study into methods of improving exercise adherence is required.

## Competing interests

The authors declare that they have no competing interests.

## Authors' contributions

The study was designed by JW, RJ and KF. Analysis was performed by JW. The first draft of the paper was written by JW and all authors provided critical input and revisions of all further drafts.

## Pre-publication history

The pre-publication history for this paper can be accessed here:

http://www.biomedcentral.com/1471-2458/12/836/prepub
